# Peer-leaders’ experiences and challenges in distributing HIV self-test kits in a rural fishing community, Rakai, Uganda

**DOI:** 10.1186/s12889-021-10804-x

**Published:** 2021-04-12

**Authors:** Joseph K. B. Matovu, Aminah Nambuusi, Rhoda K. Wanyenze, David Serwadda

**Affiliations:** 1 Department of Disease Control and Environmental Health, Makerere University School of Public Health, P.O. Box 7072, Kampala, Uganda; 2 Busitema University Faculty of Health Sciences, Mbale, Uganda

**Keywords:** HIV self-testing, Peer-leaders, Social network members

## Abstract

**Background:**

Distribution of HIV self-test kits by trained lay people in the community has resulted in increased uptake of HIV testing services among the targeted populations. However, little data exists on the experiences and challenges faced by trained lay people while distributing the kits.

**Methods:**

This qualitative study was conducted in Kasensero fishing community, Rakai, Uganda, in September 2019. We purposely selected 18 out of 34 peer-leaders that participated in a peer-led HIV self-testing intervention to participate in a post-intervention qualitative evaluation. The main intervention included identification and training of lay people in the community (‘peer-leaders’) to distribute HIV self-test kits to pre-selected members of their social network. Data for this study were collected at the end of the intervention. Data were collected on peer-leaders’ experiences in distributing the kits, challenges experienced during distribution and suggestions on how to improve peer-led HIV self-testing in typical fishing communities in the future. Data were analyzed manually following a thematic framework approach.

**Results:**

Of the 18 peer-leaders, eleven (61.1%) were aged 20–24 years while thirteen (72.2%) had secondary education. Most (*n* = 15) of the peer-leaders reported that they found it easier to distribute the kits to their social network members, with most of them distributing the kits at the social network members’ homes or at their own homes. HIV self-test kits were distributed at varying times (e.g. in the afternoon) depending on the agreement reached between the peer-leader and their social network member. A few peer-leaders reported that some of their social network members initially hesitated to accept the kits while other peer-leaders reported that they spent a ‘lot of time’ explaining the HIV self-testing procedures to some of their illiterate members. Peer-leaders argued for supervised HIV self-testing for illiterate people and the need to continuously follow-up social network members to check if they tested for HIV.

**Conclusion:**

A majority of the peer-leaders successfully distributed the kits to their social network members save for a few who experienced challenges. These findings suggest that lay people can be trained as effective HIV self-test kits distributors to improve the distribution of kits in the community.

**Supplementary Information:**

The online version contains supplementary material available at 10.1186/s12889-021-10804-x.

## Background

A recent report from the Joint United Nations Program on HIV/AIDS shows that the world is far behind in preventing new HIV infections and projects that the 2020 90-90-90 targets will be missed in most countries [1]. As we begin the new decade, with 95-95-95 targets to achieve [2], efforts should be made to ensure that no one is left behind. However, inequities in access to HIV prevention, care and treatment still exist across the globe, with young people and adult men constituting a significant proportion of the populations that are not adequately reached by the HIV prevention response [1, 2]. As a result, up to 56% of new HIV infections in sub-Saharan Africa occur among young people (15–24 years) and adult men aged 25–49 years, despite the fact that these populations constitute only 34% of the population in the region [1]. For instance, 33% of new HIV infections that occurred in sub-Saharan Africa in 2019 were among young people aged 15–24 years, although these constitute only 20% of the population in this region [1]. Similarly, although adult men aged 25–49 years constitute 14% of the population in this region, they contributed up to 23% of new HIV infections in 2019 [1]. Collectively, these findings suggest a need for implementation of innovative approaches that can reach these populations with risk-reduction HIV prevention interventions, including community-based HIV testing approaches and HIV self-testing [3, 4].

Fishing communities in sub-Saharan have been identified as areas with the highest HIV incidence and prevalence amidst low coverage of HIV services [5–7]. In Uganda, HIV prevalence in the fishing communities remains high, ranging between 17.5 to 37% [8–10] and is almost 3–6 times higher than the national average for adults which stands at 6% [11]. The high HIV prevalence in the fishing communities is as a result of an interplay of factors ranging from vulnerabilities caused by the high degree of mobility and the failure to address the low HIV knowledge, attitudes and practices that are prevalent in these communities [12, 13]. Although access to and uptake of HIV prevention and treatment services has increased in Uganda’s health facilities and at community level [14], utilization of such services remains low in the fishing communities because of limited access to health services [15–17]. Evidence shows that community-based HIV self-testing approaches, in which HIV self-test kits are distributed by trained lay people door-to-door [18, 19] or through existing social networks [20, 21], can help to increase uptake of HIV testing and influence HIV testing behavior, particularly in populations and settings that are often left out through conventional HIV testing services, including fishing communities. For instance, a study conducted in the United States of America found that an intervention in which HIV self-test kits were distributed through existing social networks reached a higher proportion of previously undiagnosed men who have sex with men, non-testers, and infrequent testers [21]. A pilot trial among fishermen in Buliisa, Uganda, found that the use of a peer model to distribute HIV self-test kits in a fishing community reached men who had not previously tested [22]. As noted by Matovu et al. [23], the use of community-based, trained lay providers, is popular because people can easily obtain kits at their convenience, given that the distributors live within the same community as the potential users. Collectively, these studies suggest that community-based HIV self-testing is highly acceptable and can increase HIV testing behavior among populations that are still missed in the HIV prevention response.

However, although community-based HIV self-testing distribution is feasible and acceptable across population and settings, few studies, if any, have explored the experiences and challenges encountered by trained lay providers during the distribution of HIV self-test kits in the community. Exploring such experiences is crucial to inform the scale-up of community-based HIV self-testing interventions but also to devise strategies necessary to address challenges that are inherent in such interventions. Our study explored the experiences and challenges experienced by peer-leaders during the distribution of HIV self-test kits to pre-selected social network members in a fishing community in rural Uganda.

## Methods

### Study design

This qualitative study was conducted at the end of a large, peer-led HIV self-testing intervention implemented to assess the feasibility and acceptability of a peer-led HIV self-testing intervention in Kasensero fishing community, Rakai, Uganda. The study methods for the main intervention are described elsewhere [24]. In brief, the intervention entailed identification of lay people (referred to as ‘peer-leaders’ in this paper) in the community who were trained to distribute HIV self-test kits to pre-selected members of their social networks. The intervention was implemented in three study communities (Kasensero landing site, Gwanda and Kyebe) that form part of Kasensero fishing community along the shores of Lake Victoria in Uganda, between July and August 2019. This post-intervention evaluation was conducted at the end of the intervention in September 2019 to explore peer-leaders’ experiences and challenges experienced in distributing HIV self-test kits and suggestions on how to improve implementation of a peer-led HIV self-testing program in a typical fishing community.

### Study population

Thirty-four peer-leaders participated in the distribution of HIV self-test kits as part of the main intervention. Of these, 18 peer-leaders (12 males and 6 female) were purposely selected to participate in this study. Each peer-leader represented a specific social network grouping. Social networks were defined as loose groupings of people who interacted on a daily basis based on the nature of their work (e.g. fishermen, boat pushers, boda-boda [motorcycle taxi] riders, sportsmen, netball players, salon owners and workers, restaurant owners and workers) or who were members of existing community groups (e.g. savings groups, talent groups). The ultimate goal was to identify and train a peer-leader who was as close as possible to members of their social network to facilitate the distribution of HIV self-test kits. We purposely invited peer-leaders with varying levels of success in distributing HIV self-test kits including those who distributed all the kits that they received; those who distributed half of the kits, and those who distributed less than half of the kits to document experiences and challenges across different HIV self-test distribution patterns.

### Peer-leaders’ selection

As already mentioned, peer-leaders were selected from existing social networks. Prior to their selection, community meetings were convened in selected locations and the qualities of a peer-leader were read to the attendants by a member of the study team. While in these meetings, participants were asked to choose one (1) peer-leader who possessed the desired qualities to represent each social network. To be a peer-leader, one had to be a permanent resident in the community, a member of an existing social network group and either aged 18–24 years (to be a peer-leader for adolescents and young people aged 15–24 years) or 25 years or older to be a peer-leader for those aged 25+ years. Peer-leaders for adolescents and young people aged 15–24 years could be male or female but peer-leaders for those aged 25+ years were exclusively males since the intervention only targeted adult men aged 25+ years. In general, the primary requirement for one to become a peer-leader was belonging to a social network group, irrespective of their occupation or HIV status. All the chosen peer-leaders were trained about HIV self-testing processes (including how to open the package containing the kits, how to obtain the oral swab, how to put the kit in the buffer solution, how to time the 20 min needed for the test, and how to read and interpret results) and asked to nominate up to 20 members of their social networks who would receive kits from them if they were found to be eligible for study enrolment.

All nominated social network members were screened for study enrolment (e.g. nominated members had to be of unknown or self-reported HIV negative status at the time of enrolment) by the study team and up to 10 of those screened were administered a baseline study questionnaire. Our focus on HIV-negative social network members was because of the need to identify first-time HIV-positives who could be linked to HIV care as soon as they were confirmed to be HIV-positive. Peer-leaders received the number of kits equivalent to the number of enrolled social network members (for a maximum of 10 kits). Social network members were instructed to contact their peer leaders to receive their kits. Peer-leaders received a pre-generated list of eligible social network members from the study team and used this to identify any of their social network members who qualified to receive the kits. Upon completing the distribution exercise, peer-leaders completed a socio-demographic form to capture details of the person that they gave kits to (including age, sex, education, marital status). The information on the completed forms was compared to the baseline records to ensure that the kits were given to the right individuals. Kits were distributed between July and August 2019, as part of the main intervention.

### Data collection procedures and methods

Qualitative data were collected using a key informant interview guide (see Additional File 1) between September 2–6, 2019. Of the 18 peer-leaders who were interviewed, nine distributed all the ten kits given to them; seven distributed between five to nine kits while two peer-leaders distributed between one to four kits. Data were collected on peer-leaders’ experiences while distributing the kits (e.g. *Tell me how you accomplished the HIV self-test kits distribution exercise; that is, tell me what happened from the time you received the kits from the study team up to the time you gave them out to your social network members*); challenges experienced during the distribution of kits (e.g. *What challenges did you experience during the process of distributing kits to your social network members? How can these challenges be minimized in the future?*) and suggestions on how to improve HIV self-testing distribution process in a typical fishing community (e.g. *If you had the opportunity to improve the process of distributing HIV self-test kits to social network members, what would you recommend?*). Interviews were conducted in Luganda, the native language spoken in the area. Data were collected by two trained Social Scientists (one with a Master of Science in Population and Reproductive Health and the other with a Bachelor’s degree in Guidance and Counseling) who were native Luganda speakers with prior experience in collecting qualitative data. Interviews lasted between 40 min and one (1) hour; they were audio-recorded with permission from the peer-leaders, transcribed verbatim and translated into English by the two Social Scientists who were engaged in data collection.

### Data analysis

We conducted manual, deductive, thematic analysis within a realist/essentialist framework. Data were analyzed at a semantic level guided by the six steps suggested by Braun and Clarke [25]. The six steps include: a) familiarizing oneself with the data; b) generating initial codes; c) searching for themes; d) reviewing themes; e) defining and naming themes, and f) producing the report. These steps were not followed in a linear fashion; instead, the process of data analysis involved moving back and forth across the different steps and, where necessary, one or more steps were considered together. Data analysis was primarily conducted by JKBM and AN who constantly met to compare the themes generated in order to compile a final list of codes that was used during the coding of all the transcripts.

Initially, JKBM and AN identified three transcripts from each study community (for a total of nine transcripts across the three study communities) and independently reviewed them to identify any meaningful patterns across data, participants, and study communities. This initial step was essential to generate insights that helped to inform the identification of codes. This process was completed through revisiting at least half of the audio-recorded interviews and comparing the transcripts against the audio-recording. Since the transcribed data were organized by the same sections as in the main study tool, we agreed to focus our initial analysis on key questions that inquired into the main overarching (a priori) themes (i.e. experiences during the distribution of kits; challenges experienced during the distribution of kits, and suggestions for improving the implementation of a peer-led HIV self-testing intervention in a typical fishing community). Four questions (one for each overarching theme) were selected to guide this initial analysis. For each question selected, we read through the transcripts and coded chunks of text that alluded to each main overarching theme. The coded chunks of text were then copied and pasted into a generic matrix (created in MS Excel), arranged by the overarching themes. The codes generated through this process were then applied to the remaining transcripts to complete the coding process. The completed matrix was then reviewed by JKBM and AN to identify any emerging themes and sub-themes, and the best quotations that supported a priori and emerging themes and sub-themes. Where we identified different quotations in support of a particular sub-theme; this was resolved through discussion to reach consensus on the quotations to use in reporting the findings. KII identifiers are used in the place of peer-leaders’ names. Study findings are presented in conformity with the consolidated criteria for reporting qualitative studies [26].

## Results

### Peer-leaders’ characteristics

Table 1 shows the characteristics of the 18 peer-leaders who were enrolled in this study. Of those interviewed, six (33.3%) were from Kasensero landing site, six (33.3%) were from Gwanda and six (33.3%) were from Kyebe. In each community, approximately 67% of the peer-leaders were males. A majority of the peer-leaders were aged 20–24 years (*n* = 11; 61.1%) while 72.2% (*n* = 13) had secondary education. All the peer-leaders had ever tested for HIV before the intervention; 50% (*n* = 9) distributed all the 10 kits that they received.

**Table 1:** Characteristics of the peer-leaders by study community of residence

Characteristics	Study communities		
	Kasensero	Gwanda	Kyebe
**Sex**			
Female	2 (33.3)	2 (33.3)	2 (33.3)
Male	4 (66.7)	4 (66.7)	4 (66.7)
**Age-group**			
18–19	1 (16.7)	0 (0.0)	0 (0.0)
20–24	1 (16.7)	6 (100)	4 (66.7)
25+ years	4 (66.7)	0 (0.0)	2 (33.3)
**Education**			
Primary education	1 (16.7)	3 (50)	1 (16.7)
Secondary education	5 (83.3)	3 (50)	5 (83.3)
**Prior testing Experience**	6 (100)	6 (100)	6 (100)
**Number of kits distributed by the peer-leader**			
1–4	0	2 (33.3)	0
5–9	3 (50)	2 (33.3)	2 (33.3)
10	3 (50)	2 (33.3)	4 (66.7)

### Experiences, challenges and suggestions to improve the HIV self-testing program

The results presented in this paper are organized around three overarching themes: a) peer-leaders’ experiences, b) challenges faced by peer-leaders during the HIV self-test kits distribution exercise and c) suggestions to improve implementation of a peer-led HIV self-testing program in a typical fishing community setting, as shown below.

- Peer-leaders’ experiences while distributing HIV self-test kits

Peer-leaders’ narratives of their experiences in distributing the kits pertain to three important aspects: the setting/venue of the HIV self-test kits distribution event; timing of the HIV self-test kits distribution event, and requests for assistance from social network members in performing the self-test, as shown in the following sub-sections:

i) *Setting/venue of the HIV self-test kits distribution event*

When asked where they distributed the kits from, most peer-leaders (15) reported that they distributed the kits from their homes or from the homes of their social network members. Peer-leaders said that these were safe and private spaces for people to receive kits from without anyone else noticing, since there is a lot of stigma still attached to HIV testing. Homes also provide a conducive environment for peer-leaders to explain the HIV self-testing process to their social network members and to address any questions that arise.

*“That is the good place because the person that you have found at home can get it [kit] and then keep it in his home. The person who finds you at your home takes it as an important issue. He will get it, take it home and test. If you find someone along the way or in town and tell him that X, stop there. When you give him the kit, he might be holding other things; he will not consider it as important. It would be good to find the person at home or in your home”* [Peer-leader of a drummers’ group, Kyebe]*.*

The remaining three peer-leaders, whose social network members were male youth, gave out kits from other places within the community. This is because some of the youth did not have permanent residences or locations where they could be located. A peer-leader of the *Mukene* (silver fish) sellers’ group in Kasensero reported that he met some of his social network members at his friend’s library while he met others at the lake shore while they were laying fishing nets. He had earlier told his social network members to collect their kits from his home but they seemed adamant. He claimed that he found it challenging to give out kits to his social network members because they were recommended to him by a member of the village health team. As a result, he spent quite some time convincing them to use the kits. Another peer-leader of a salon group in Gwanda gave out all the HIV self-test kits to his social network members from the salon because it’s where they spend their free time.

A peer-leader of the games group in Kyebe gave out kits from the pool table. He would call his members aside and request them to spare some time for him so that he could explain the HIV self-testing procedures to them. Out of curiosity, those who he wasn’t supposed to give kits to requested him to show them what the kit looked like which he did.

*“ … The good thing is that, that person was around. I told him to spare a ‘minute’. I put him aside and told him that I [have] got your kit. The other people at the pool table wanted to come and see. I let them see the kit and then they went back afterwards* [Peer-leader of a games’ group, Kyebe].

When peer-leaders were distributing the kits especially in the homes of their social network members, they found some of the partners of their social network members at home. These partners hadn’t been registered to take part in the HIV self-testing program. Peer-leaders had to explain to these partners what had brought them at their social network members’ homes. A peer-leader in Kyebe narrated what transpired when he went to the homes of his social network members to give them kits and found their partners at home.

*“When I reached there, some of them are married. I was given kits of only men so the women used to quarrel and said that how can you only give this one, I also want to test myself to know my status. I would tell them that I was given kits for men only but not for women but next time, if it happens that they give us kits, we shall give you also … ”* [Peer-leader of a footballers’ group, Kyebe].

Women who belonged to a savings group in Kyebe were worried of receiving kits at their homes because of fear that their partners might find out that they were going to test for HIV. When they contacted the peer-leader, she advised them to inform their partners about their involvement in the HIV self-testing program to avoid problems that might arise if their partners found out about their involvement in the HIV self-testing program, as indicated in the quotation below:

*“Some of the members said that what if our spouses get to know. I told them that you should tell your husband before you leave that I am going to X’s place to discuss this program so I will explain to you what transpires. I told them that they should explain to those people what is going on.* [Peer-leader of a savings’ group, Kyebe].

ii) *Timing of the HIV self-test kits distribution event*

Peer-leaders gave out kits at different time intervals depending on the nature of work that their social network members were engaged in. Most peer-leaders preferred to give out kits in the afternoon from 2 pm or in the evening after 5 pm when their social network members were free after completing the day’s work.

*“He would have finished doing all the things that worry him. He will have finished work at 2pm. We go to the playground at 3 to 4pm and we are done by 5pm so I can put him aside and explain to him that I brought the kit. You then give the kit and explain to him how it works* [Peer-leader of a sports’ group, Gwanda].

One the one hand, peer-leaders of the farmers’ group preferred to give out kits between 11 am and 12 pm or later in the evening when they were free. This is because most farmers usually leave their gardens by 11 am and head home directly. It was easier to talk to them when they were done with their work. On the other hand, the peer-leader of *boda-boda* riders was comfortable giving out kits at any time. Because of the nature of their work, *boda-boda* riders aren’t sure of when they can be free so they hang out at their stage at different time intervals.

*“Since I represent boda-boda riders, I spend a lot of time on the road so I can give it out any time. I can explain to someone as we hang out at the stage. I can’t tell you the exact time because if I told you that I will do it in the evening, evening will come when I am very busy yet I may not be busy in the morning. So, I can give out kits anytime”* [Peer-leader of a boda-boda riders’ group, Gwanda]

iii) *Requests for assistance from social network members in performing the self-test*

A central tenet of the peer-led HIV self-testing intervention was the emphasis on unsupervised HIV self-testing; i.e. social network members were expected to perform the self-testing procedure on their own, without any external assistance. Peer-leaders reported that, on the whole, most of their social network members were able perform to the self-test unassisted. However, peer-leaders reported that a few members asked them for assistance while conducting the HIV self-test. Most of those who asked for support were afraid of the likely HIV test results while some of them could not correctly estimate the 20 min needed to read the results since they didn’t have a phone or watch. Some of those who requested for assistance reported that they had forgotten the HIV self-testing procedures. Other peer-leaders agreed to assist their social network members because they thought that they (social network members) might benefit from additional psycho-social support, especially after a positive result.

*“The other thing is that when someone tests positive, I, the peer-leader, can help her by taking her to the health center which will re-test her. I shouldn’t [leave] members alone because a person might test in my presence and then she feels weak. You told us that if someone tests and she is not sure of her results, she has to go and see the health worker but she might not go there because her/his friends will laugh at him/her. He/she can say that let me die and they bury me. It’s very helpful if I am around. I will give him/her support, counsel him/her then take him/her to the health worker to explain to him/her what else needs to be done”* [Peer-leader of DREAMS^1^ trainees].

Some social network members requested their peer-leaders to allow them to conduct the test from their (peer-leaders’) homes. Notably, a peer-leader of a women savings group narrated that some women weren’t comfortable with their spouses finding out that they had tested without their knowledge and that is why they chose to test from the peer-leaders’ homes to avoid conflicts with their spouses.

*“I called them and they told me that it would be good for us to come to your home because we aren’t sure of our spouses. If he finds you testing yet you didn’t inform him, do you realize that he might get them and throw them away. That might bring about problems. Some said that X, we would want to do this thing with you around. We can’t go to someone else’s home to do that. I had to bring those people to my home … Some members said that X, some of us don’t have a phone or a watch, some of us don’t know how to count, it would be good for us to come and you estimate the time for us. I told them that I wouldn’t want to know your results. I want you to test from your home. But they said that we aren’t going to show you our results, we just want you to direct us and estimate the time for us and we do our work”* [Peer-leader of a savings’ group, Kyebe].

This peer-leader told us some of the women in the savings group only told their male partners about their involvement in the HIV self-testing program after they had already used the kits to self-test for HIV. This did not amuse the male partners, preferably because they had not consulted them before joining the program. The male partners decided to talk to the peer-leader about it.

*“ … Some of their spouses came to me and told me that what have you given to our wives, are they good things or they will cause problems. I explained to their husbands and told them that these things are good. Your wife might come and tell you to use the kit because you don’t want to go to the health center to know your status. Some men accepted it so the women are now free. If I give them kits, they can go home and test without any problem”* [Peer-leader of a savings’ group, Kyebe].

b) Challenges faced during the distribution of HIV self-test kits

Peer-leaders experienced a few hiccups that affected timely distribution of kits. These include; a) structural factors underlying the HIV self-test kits distribution process, b) hesitancy to obtain kits from peer-leaders and c) factors impacting HIV self-test kits use, as the following sub-sections illustrate.

i) *Structural factors underlying the HIV self-test kits distribution process*

The distribution of HIV self-test kits was affected by a number of structural factors including social network members not being found at home, lack of correct addresses and conflicting work schedules. Three peer-leaders felt that they spent a ‘lot of time’ looking their social network members in the community to give them kits. This meant that they either had to wait for them to return from wherever they would have gone or come back again to be able to give them kits. Some peer-leaders found themselves visiting the homes of their social network members several times to be able to give them kits.

*“The challenge I experienced is that I would go to someone’s place to take the kit but when he is not around. He went to this place and you have to wait for him to come back and if he comes to town, you tell him that I have your kit that the health worker told me to bring to you. He tells you that you wait, I am in a hurry but I will come back. He refuses to come back and you have to look for him”* [Peer-leader of a games’ group, Kyebe].

ii) *Hesitancy to obtain HIV self-test kits from peer-leaders*

Since HIV self-testing is still a new HIV testing strategy in most of the study communities, some social network members were hesitant to obtain the kits from their peer-leaders. A peer-leader of the *Mukene* [silver fish] sellers’ group reported that he found difficulty convincing three of his social network members to take their kits although they had initially indicated that they would take them. There were fears among some youth that they were being used as trial subjects in the HIV self-testing program; so, whenever they were contacted, they pretended to be busy with work. Peer-leaders had to provide a little more explanation about the study before they convinced them to take their kits.

*“The only challenge I got was to explain to them a lot about that kit. You know the youth; you can talk to him as he pretends to be busy and is trying to make money”* [Peer-leader of a Mukene [*silver fish*] sellers’ group, Kasensero].

iii) *Factors impeding HIV self-test kits use*

To be able to use the kits successfully, it is imperative that users know how to read and write – at least they should be able to tell the time since they need to time the HIV self-testing process. This is because the HIV self-test kits package comes with user instructions (including pictorial illustrations) in English as well as in other local languages to help users perform the test on their own. However, some peer-leaders reported that some of their social network members had difficulties understanding the HIV self-testing procedures. This was particularly the case with social network members who were illiterate. As a result, peer-leaders spent a ‘lot of time’ explaining the HIV self-testing procedures to their members. Unfortunately, some members failed to grasp how to conduct an HIV self-test and requested peer-leaders to help them while they were performing the test.

“*All the members that we educate don’t understand at the same rate, some of them disturbed us. You would educate him but he still feels that there is something he hasn’t understood so you had to spend a lot of time educating him”* [Peer-leader of a sports’ group, Gwanda]*.*

In one particular case, a peer-leader took a lot of time explaining to her social network member how the self-test is done only to realize later that she had not conducted the test correctly. This social network member was later given another kit to repeat the HIV self-testing process:

“*There is someone who experienced that. I called her when she was coming from her home, I told her that I need to see her … I went with the kit to her home and explained to her various times. I would tell her to explain to me what I had told her but she would explain while missing some things. She didn’t understand many things... Afterwards she told me that she had understood and I told her that after using the kit, she should take it directly to the health worker. After a few days, I asked her that did you see the health worker after using the kit and she told me that aah … I used the kit but I got wrong results. She didn’t say anything else … I didn’t know if I could help her, for example, by taking her to counselor X to get another kit so that she could perform the test from there. Afterwards, she repeated the test when you [members of the study team] came back [for the follow-up visit]* [Peer-leader of DREAMS trainees, Kasensero].

c) Suggestions for improving the implementation of a peer-led, HIV self-testing intervention in a typical fishing community

We asked peer-leaders what they would do to improve the implementation of a peer-led, HIV self-testing intervention within a typical fishing community. In response, peer-leaders made a number of suggestions ranging from the need to allow for supervised HIV self-testing for illiterate members of the community; the need to follow-up social network members to check if they have used the kit; and the need to choose peer-leaders who are humble, approachable and who can keep secrets. The following sub-sections provide insights into what peer-leaders thought could help to improve the implementation of a peer-led, HIV self-testing intervention in a fishing community in the future.

i) *Allow for supervised, peer-led HIV self-testing, where appropriate*

Peer-leaders indicated that some of their social network members were illiterate and could not accurately follow the HIV self-testing user instructions. Other peer-leaders reported that their members could not accurately estimate the 20 min needed to perform the self-test or that they needed emotional support from someone in case they tested positive. For that reason, some peer-leaders suggested a need to allow for supervised HIV self-testing in which a peer-leader would be physically present to support their members in performing the HIV self-test. When peer-leaders were asked how they dealt with their illiterate members, some of them reported that they actually helped them to complete the HIV self-testing process even if they (the peer-leaders) were not meant to do this, as intimated by a peer-leader of a footballers’ group:

“*That same thing happened to me. I explained to my members that this is an HIV self-testing kit. You are the one supposed to test yourself and no one is supposed to help you. Three of my ten members said … we don’t know how to read time and we might make mistakes. I told them what to do but out of the three its one who accepted and tested himself. With the other two, I made a mistake but I helped them. The health workers told us that we aren’t supposed to help people test. They should test themselves and understand but they requested me and forced me to do it”* [Peer-leader of a footballers’ group, Kyebe].

ii) *Need to follow-up social network members to ensure that they have used the kit*

Peer-leaders suggested the need to follow-up their social network members after giving them kits to ensure that they have used them to test for HIV. This is because some of the social network members who test positive might fail to disclose their status to anyone, or if they got HIV treatment, they may fail to take their medicines. Following up the social network members who have taken the kits home could also help to support those who test HIV-positive to link to HIV care and/or continue with their medication as the following quotation illustrates:

*“Follow up on people who test positive. Someone will go and get medicine for the first time and he/she experiences some side effects and says that I won’t go back”* [Peer-leader of a talents’ group, Gwanda].

iii) *Choose peer-leaders who are humble, approachable and who can keep secrets*

In order to improve the implementation of a peer-led HIV self-testing program in the future, any selected peer-leaders should be humble, easily approachable and be able to keep secrets. With those qualities, the social network members can easily trust them and also open up to them about anything. Having such people distributing kits will enhance the smooth distribution of kits in the community.

*“Peer-leaders should be educated on how to handle people well. You can’t come from wherever and bump into people. You have to come and tell them that I have information in a humble way. You can call your friends who you play football with. After playing for 30 minutes, you can tell them that let’s use these 20 minutes to talk about this program. You can then talk about that program”* [Peer-leader of a farmers’ group, Gwanda]*.*

## Discussion

This qualitative study discusses the experiences and challenges experienced by peer-leaders while distributing HIV self-test kits to members of their social networks in Kasensero fishing community, Rakai district, Uganda. Our findings show that: a) most of the peer-leaders found it easy to distribute the kits and that the distribution event mainly took place at the social network members’ homes or at the homes of the peer-leaders; b) peer-leaders distributed kits at different times depending on the nature of the social network grouping; and c) although our intention was to promote unsupervised HIV self-testing, some peer-leaders indicated that they assisted their social network members to self-test for HIV. A few peer-leaders experienced some challenges: a) initial hesitation by some social network members to pick their kits; b) spending a lot of time looking for social network members in the community to give them kits and c) taking time explaining the HIV self-testing procedures to some illiterate members of their social networks.

Our finding that peer-leaders preferred to distribute kits from their homes or at the homes of the social network member is not surprising given the discrete nature of the HIV self-testing process. Peer-leaders indicated that their social network members found it convenient to receive their kits at venues that ensured privacy and the peer-leaders’ homes or the social network members’ homes were found to meet these qualities. Other studies done in Zimbabwe and Kenya also found that people preferred home delivery of kits to ensure privacy [27, 28]. Analysis from a study conducted in the United Kingdom highlighted the need to have privacy in order to discuss and facilitate the undertaking of HIV self-testing and avoid stigma among black African people [29]. There is need for people who self-test for HIV to have control over their own information. Future studies should ensure privacy while giving out kits so that people who test for HIV aren’t affected by the stigma that is attached to HIV testing. Assurance of privacy will increase on the number of people who are willing to self-test and know their HIV status.

We found that peer-leaders distributed the kits at different times with regard to the time when their social network members returned from work. Peer-leaders found it convenient to give kits to social network members after they had finished the days’ work and were free to receive their kits from the peer-leaders. We suggest that future interventions should include asking social network members about their preferred time of receiving the kits. Although our intention was to promote unsupervised HIV self-testing, some peer-leaders mentioned that social network members requested them to be present at the time of testing. Peer-leaders reported that their social network members needed counseling and support in performing HIV self-testing which was new to them. Illiterate social network members needed peer-leaders to help them estimate the time needed to read the test results and to interpret for them the HIV self-test results. These findings suggest a need to integrate peer-supported initiatives within peer-led HIV self-testing interventions to support HIV self-test kits users who may not be able to perform the self-test on their own.

Our findings show that while peer-leaders described largely positive experiences, a few of them experienced some challenges while distributing the kits. Notably, some people were hesitant to pick their HIV self-test kits from them at first thinking that they had been recruited as study subjects. This aligns with what has been documented in other studies like one that was conducted among African American and Latino men who have sex with men where some peers-leaders also experienced initial resistance from friends when they heard that it was a study [21, 30]. In our study, peer-leaders had to explain the importance of the social network members knowing their HIV status as that is the first step into HIV treatment and care and emphasized the fact that social network members were not research subjects.

We obtained useful suggestions on how best to improve the use of peer-leaders to distribute HIV self-test kits in a typical fishing community. These included the need to allow for supervised HIV self-testing for illiterate members, the need to follow-up social network members to ensure that they have used the kits and the need to choose peer-leaders who are humble, approachable and who can keep secrets [31]. The need for supervised HIV self-testing for illiterate members of the community can be justified on the ground that illiterate people are not able to read user instructions and can thus fail to perform the test correctly. Even if the HIV self-test kits package can also include user instructions in the local language, their use can only be ensured if people are able to read in their local language. Thus, it might be helpful to train lay providers in the community (e.g. members of village health team or community health workers) in HIV self-testing procedures to support illiterate members of the community to perform HIV self-testing procedures with minimal, if any, challenges. Findings from our study further suggest the need to follow up social network members to ensure that they have used the kit. Peer-leaders should contact their social network members after giving them kits to find out if they were able to use the kits and help those who weren’t able to. A study conducted among men who have sex with men in Uganda [32] also found that in order for peer distribution strategies to remain acceptable, there is a need for a follow up strategy to re-emphasize the importance of confirmatory testing for those who test positive. The need to follow up social network members was one of the suggestions mentioned for improving the HIV peer-led distribution strategy. This helps ensure that all the people who were given kits use them to test themselves and get to know their status. Participants from a study conducted in Tanzania [33] also mentioned the need to follow people who are given kits as failure to do so may lead to delayed initiation into care among HIV-positive participants.

Our study findings have implications for the delivery of HIV self-test kits in other settings beyond Kasensero fishing community, especially in populations and settings where provision of conventional HIV testing services is limited by people’s mobility patterns or the nature of occupations. For instance, the use of peer educators has been found to be acceptable among sex workers in Uganda and Zambia [34, 35] and evidence shows high levels of acceptability of HIV self-testing among sex workers reached through peer educator groups. In another study in Zambia and Malawi [18, 19], the use of trained lay counsellors, also known as Community HIV Care Providers, led to increased uptake of HIV testing services particularly among population groups that were always not found at home, including men. As similar interventions are scaled-up across populations and settings, the use of trained lay providers will become normative and play an essential role in the distribution of HIV self-test kits to hard-to-reach and highly mobile populations including those living in the fishing communities across Africa. We believe that our study findings will inform program planners and policy makers in the areas that require additional strengthening to ensure successful peer-led HIV self-test kits distribution while addressing the challenges inherent in using this approach.

### Study limitations and strengths

In our study, one peer-leader was not popular in their group and that is why some of the social network members found it difficult to reach out to them to be able to collect their kits. However, we noted that this peer-leader hadn’t lived in the community for a long time. Secondly, the distribution of HIV self-test kits was not monitored so we can’t verify the experiences and challenges reported. These are only self-reported. In addition, the experiences and challenges reported may not represent what each and every peer-leader experienced. The experiences might be for those who were interviewed and it is likely that if we interviewed another set of peer-leaders, we could have obtained different experiences. Despite the above mentioned limitations, our study had the following strengths. This is the first study to evaluate the experiences and challenges of distributing kits which provides an avenue to use them in the future. In addition, our study had peer-leaders with varying successes in distributing kits which represents what will happen in reality. We believe that the findings of this novel, peer-led HIV self-testing intervention will provide insights into how similar programs can be implemented with minimal challenges to enhance HIV testing and linkage to care among populations that are usually missed through conventional HIV testing programs.

## Conclusion

Our findings show that peer-leaders distributed the kits successfully without experiencing any major challenges with the exception of a few of them who reported challenges relating to providing more time to their social network members to understand the HIV self-testing process and with members who were hesitant to pick their kits. Peer-leaders recommended for supervised HIV self-testing for illiterate members to reduce HIV self-testing errors and enhance accuracy. Peer-leaders should also follow up their network members to ensure that they use the kits to self-test for HIV and those who test HIV-positive initiated on antiretroviral therapy. In general, our findings suggest that using lay people to distribute HIV self-test kits is effective in increasing the uptake of HIV self-testing in typical fishing communities. Future studies should ensure privacy while distributing kits and studies should consider having supervised HIV self-testing for illiterate members.

## Supplementary Information

**Additional file 1.** Study Tool. Key Informant Interview Guide

## Data Availability

The datasets generated and/or analysed during the current study are not publicly available as they cannot be completely anonymized. They are available from the corresponding author on reasonable request.

## Footnotes

1 The DREAMS (Determined, Resilient, Empowered, AIDS-free, Mentored and Safe) partnership is an initiative of the United States President’s Emergency Plan for AIDS Relief (PEPFAR) intended to reduce rates of HIV infection among HIV-negative adolescent girls and young women (AGYW) in the highest HIV burden countries. In sub-Saharan Africa, the DREAMS initiative is implemented in 10 countries including Kenya, Lesotho, Malawi, Mozambique, South Africa, Swaziland, Tanzania, Uganda, Zambia and Zimbabwe.
