# Supplementary material for: Peer-leaders’ experiences and challenges in distributing HIV self-test kits in a rural fishing community, Rakai, Uganda
Source: BMC Public Health. 2021 Apr 12;21:708. doi: 10.1186/s12889-021-10804-x (PMC8042983; doi:10.1186/s12889-021-10804-x)
Supplement: Supplementary file 1 — Additional file 1. Study Tool. Key Informant Interview Guide [file 12889_2021_10804_MOESM1_ESM.doc]

**Peer-leaders’ experiences and challenges in distributing HIV self-test kits in a rural fishing community, Rakai, Uganda**

Joseph KB Matovu^1,2*^, Aminah Nambuusi^1^, Rhoda K. Wanyenze^1^, David Serwadda^1^

^1^Makerere University School of Public Health, Kampala, Uganda

^2^Busitema University Faculty of Health Sciences, Mbale, Uganda

**KEY INFORMANT INTERVIEW GUIDE**

[FOR **PEER-LEADERS**]

**FOR OFFICIAL USE ONLY**

**Tracking Number**: Please generate the tracking number using the format: ***KII/KAS-PL/19/08/20/01*** where KII= Key Informant Interview [Method]; **KAS** = KASENSERO (Replace with **GWA** for GWANDA or **KYE** for KYEBE) [community name]; the ‘**PL**’ denotes “Peer-Leader”; 19/08/20 – Date of Interview (in the order **yy/mm/dd**) and 01 – first KII interview.

Moderator #: ___________________________

Tracking #: ______________________________

Date of interview: ______/ _______/ ____________

Community Name: _______________________________

Venue: ___________________________

Language of interview: ________________________

Time started: ________________ Time ended: _________________

**SOCIO-DEMOGRAPHIC CHARACTERISTICS OF PARTICIPANTS**

1. Sex

Male 1

Female 2

1. Educational level

No education 1

Primary education 2

Lower secondary (S1-S4) 3

Upper secondary (S5-S6) 4

University 5

Other level _______________________ 6

1. Social group network: _______________________
2. Prior HIV testing experience

Ever-tested 1

Never tested 2

1. Category of peer-leader by how many kits they distributed

Gave out all kits to peer-leaders 1

Gave out half or more but less than 10 kits (5-9 kits) 2

Gave out less than half of the kits (1-4 kits) 3

**SECTION A: GENERAL QUESTIONS**

1. Let’s start by talking about HIV self-testing: How would you describe the process of HIV self-testing to someone who is not familiar with it?
2. In general, if you wanted to give a kit to someone, what is the best way of approaching them? Probe: what would be the best time to give them the kit? Which place is easier for you to give them the kit?

**SECTION B: HIV SEF-TEST KITS DISTRIBUTION EXPERIENCE**

1. How many kits did you receive? Of these, how many kits did you distribute to members of your social network? Were you able to distribute all the kits or some of the kits? If you distributed some of the kits, how many kits did you distribute? What made you fail to distribute all the kits to all your social network members? If you gave out all the kits as expected, what made it easy for you to distribute all the kits to all your social network members?
2. Tell me how you accomplished the HIV self-test kits distribution exercise; that is, tell me what happened from the time you received the kits from the study team up to the time you gave them out to your social network members.
3. When you gave the kits to your social network members, what did you tell them the kits were specifically meant for? How did you explain the HIV self-testing process to your social network member?
4. Where did you meet the social network members at the time of distributing the kits? Was this a suitable venue or would you rather that you should have given them out at another place? If you think that another place would have been better; please explain why you think so.

**SECTION C: HIV SELF-TESTING OF THE SOCIAL NETWORK MEMBER**

1. Some peer-leaders assisted their social network members to perform HIV self-testing. What is your opinion about this, considering that the process requires one to self-test themselves? How else could these peer-leaders have assisted their social network members to test for HIV?
2. Some people believe that peer-leaders should have stayed around to give support to their social network members as they performed HIV self-testing. What is your opinion about this? If you were the one receiving the kit from your peer-leader, would you have minded if the peer-leader stayed on to offer you support?
3. Did you get to know your social network members’ HIV results? If yes, how did you manage to know these results? Probe: were you told by the social network member? Did you perform the self-testing exercise on behalf of your social network member, and, by default, saw his/her results? Did the social network member give you their used kit to take to the health facility?

**SECTION D: PEER-LEADERS’ EXPERIENCE IN HIV SELF-TESTING**

1. What has been your experience as a peer-leader distributing HIV self-test kits to other people in the community? Would you say the process would have been easier if the kits were left at the health facility and peer-leaders just encouraged their social network members to go and pick them from there?
2. What challenges did you experience during the process of distributing kits to your social network members? How can these challenges be minimized in the future?
3. Let’s talk about the peer-leaders’ facilitation during the HIVST distribution exercise: how else could peer-leaders have been facilitated to do their job better?
4. How can the process of peer-leaders distributing kits to members of their social network be improved to reach more people with HIV self-test kits?

**SECTION E: ENDING QUESTIONS**

1. People who test HIV-positive should seek confirmatory HIV testing at existing health facilities and be linked to HIV care if they are confirmed to be HIV-positive. However, sometimes people fail to seek confirmatory HIV testing or link to HIV care. What should be done to improve the proportion of people who seek confirmatory HIV testing?
2. In most cases, people who self-test HIV-positive do not go to the health facility to initiate HIV treatment. This suggests a need for alternative options to improve ART initiation. Please comment on the following options with regard to improving ART initiation among people who self-test HIV-positive in the community:
3. Home-based initiation, by a nurse from the health facility
4. Home-based initiation, by a trained peer-leader
5. Home-based initiation, by a trained HIV counselor or expert client in the community
6. Initiation through an existing community ART group in the community
7. If you had the opportunity to improve the process of distributing HIV self-test kits to social network members, what would you recommend? Please explain your response.

END OF INTERVIEW

THANK YOU FOR YOUR TIME
